# Supportive University Climate and AI Dependence Among Undergraduates: The Sequential Mediating Role of Academic Self-Efficacy and Self-Control

**DOI:** 10.3390/bs16060902

**Published:** 2026-06-02

**Authors:** Jinfeng Lu, Weiwei Li, Yangyang Mo, Min Ge

**Affiliations:** 1 School of Education Science and Technology, Nanjing University of Posts and Telecommunications, Nanjing 210023, China; 1024163005@njupt.edu.cn (J.L.);; 2 Faculty of Education, East China Normal University, Shanghai 200062, China; 3 School of Electrical and Information Engineering, Jiangsu University, Zhenjiang 212013, China

**Keywords:** supportive university climate, AI dependence, academic self-efficacy, self-control, undergraduates

## Abstract

With the increasing incorporation of artificial intelligence (AI) into educational contexts, undergraduates’ AI dependence has become increasingly apparent, constituting a significant concern in educational psychology. Using survey data from 1142 Chinese undergraduates, this study adopts a mediation-based analytical framework to investigate the association between supportive university climate and AI dependence, with academic self-efficacy and self-control as hypothesized mediators. The findings reveal a significant negative association between supportive university climate and AI dependence. Furthermore, the data support independent and sequential mediating pathways linking supportive university climate with undergraduates’ AI dependence via academic self-efficacy and self-control. The findings suggest that a more supportive university climate is associated with higher academic self-efficacy and greater self-control, which are, in turn, linked to lower levels of AI dependence.

## 1. Introduction

Recently, the application of AI in higher education has grown rapidly, with tools such as ChatGPT and Claude becoming increasingly integrated into students’ learning processes. This growth has coincided with an emerging pattern of AI dependence, particularly among undergraduates (Fieldhouse, 2025; Singer-Freeman et al., 2025). As learners navigating a critical developmental stage, undergraduates encounter numerous challenges such as intensified academic competition, employment-related anxieties, and complex social dynamics. These factors may be associated with their elevated level of dependence on AI (X. Liu et al., 2026). International evidence suggests that AI tools have become pervasively integrated into undergraduates’ academic routines. A survey of German undergraduates found that 63.4% had used AI for academic support, with 25.2% reporting frequent use of such tools (Von Garrel & Mayer, 2023). Similarly, 37% of undergraduates in the UK report regular or frequent use of AI technologies (Arowosegbe et al., 2024). In China, a survey of over 3000 undergraduates across 13 universities found that 40.49% “frequently” and 12.29% “always” used generative AI for learning assistance (S. Wang & Huang, 2024). While frequent use does not inherently constitute pathological dependence, the pervasive adoption of AI tools raises concerns that a subset of students may develop a continual and compulsive psychological reliance—that is, AI dependence. Indeed, approximately 32% of undergraduates exhibit such dependence, a rate considerably higher than that observed in the general population (Almassaad et al., 2024). In Jordan, 36.7% of undergraduates exhibit moderate AI dependence, while 21.2% demonstrate high dependence (Al Mashagbeh et al., 2025). S. Feng and Zhou (2025) further reported that over half of Chinese students exhibit AI dependence, with dependence in daily coursework reaching 64.10%. This trend has drawn increasing research attention, as overreliance on AI may be negatively linked to the formation of creativity, emotional competence, and autonomous problem-solving, thereby posing risks to the quality of higher education (Vieriu & Petrea, 2025; Gerlich, 2025; OECD, 2026). Therefore, examining the underlying mechanisms of AI dependence among undergraduates is critical for developing effective prevention and intervention strategies.

In China, the comprehensive implementation of the “AI + Education” strategy has coincided with the widespread incorporation of AI tools into undergraduates’ daily learning environments. In this context, universities—functioning as primary environments for student learning and development—position supportive university climate as a key contextual factor associated with undergraduates’ psychological responses to technology use (Wei et al., 2025; Berhanu & Sewagegn, 2024). Supportive university climate refers to the psychosocial environment characterized by teacher support, student–student support, and opportunities for autonomy, which is associated with relatively stable and lasting influences on students’ psychological processes and behaviors (Kang et al., 2025). Previous research indicates that a supportive university climate is associated with healthier learning attitudes and more adaptive behaviors, and may also be connected to reduced levels of AI dependence (Chan & Hu, 2023). University climate, however, is not invariably supportive. In some institutional contexts, an overemphasis on academic performance and competitive rankings may be associated with a utilitarian climate in which students tend to view AI tools as a shortcut to academic advantage (Z. Yang et al., 2025). By contrast, the present study focuses specifically on the supportive dimensions of university climate and examines their association with AI dependence. The competitive or pressure-oriented facets, though theoretically important, lie beyond the empirical scope of this investigation and present a valuable direction for future research. Although previous studies suggest that a supportive university climate may be inversely related to technology dependence, the relationship between supportive university climate and undergraduates’ AI dependence within the Chinese academic context remains insufficiently examined and warrants further empirical investigation. More importantly, the underlying psychological mechanisms connecting these variables remain inadequately understood. Academic self-efficacy and self-control, as central components of individuals’ internal psychological systems, are likely to function as important mediators in this relationship. Academic self-efficacy is defined as individuals’ confidence in their capacity to complete academic tasks (Bandura, 1977; Shi et al., 2025). Students with higher academic self-efficacy tend to rely on their own capabilities to overcome academic challenges; accordingly, academic self-efficacy may be inversely associated with dependence on external AI tools (Y. Wang & Xu, 2026). Self-control denotes the capacity to regulate one’s emotions and behaviors in line with social expectations or personal standards (Nilsen et al., 2020). It plays a vital part in decision-making quality and behavioral persistence when confronting temptation, and constitutes a key internal factor in understanding and addressing college students’ technology dependence (Pamuk, 2026; Yildiz Durak et al., 2026). Therefore, incorporating individual psychological characteristics closely linked to AI dependence, namely, academic self-efficacy and self-control, is essential for clarifying the specific pathways and internal mechanisms linking supportive university climate to undergraduates’ AI dependence.

In summary, this study concentrates on the higher education context in China. It aims to systematically investigate the mechanisms linking supportive university climate, academic self-efficacy, self-control, and AI dependence among undergraduates. Specifically, this study proposes a sequential mediation model and empirically examines it using survey data gathered from Chinese undergraduates to investigate the relationship between supportive university climate and AI dependence. It specifically focuses on the sequential mediating roles of academic self-efficacy and self-control. The findings aim to deepen the academic community’s theoretical understanding of the associations between supportive university climate and AI dependence among Chinese undergraduates, as well as to offer empirical evidence to assist universities in promoting the judicious use of AI tools and in formulating relevant intervention measures.

## 2. Literature Review and Research Hypothesis

### 2.1. Supportive University Climate and AI Dependence Among Undergraduates

The concept of “supportive university climate” originates from organizational climate theory (Anderson, 1982). In the 1960s, Halpin applied the concept of organizational climate to educational research (Thomas, 1976). Since then, school climate has been explored from diverse perspectives and established as an independent field of study. As research has progressed, scholars have increasingly emphasized students’ subjective experiences, conceptualizing school climate as a psychological construct from a social-psychological perspective. Accordingly, this study defines supportive university climate as undergraduates’ perceptions of the extent to which their campus environment provides emotional, instrumental, and decision-making resources, which are stably and persistently associated with their cognition, emotions, and behaviors. Early studies, grounded in organizational sociology, classified school climate into two core dimensions: social order and social behavior (Hoy, 1990). Extending this macro-level framework, Cohen et al. (2009) further operationalized school climate into three micro-level components: teacher support, student–student support, and opportunities for autonomy. Collectively, these three dimensions reflect the emotional, instrumental, and decision-making resources that an institutional environment affords students, and are thus widely regarded as the supportive facets of school climate (Y. Jia et al., 2009; Kang et al., 2025). The present study operationalizes supportive university climate through this three-dimensional framework.

In educational psychology, AI dependence has been conceptualized from behavioral, cognitive, and integrative perspectives (Tamrin et al., 2024; Uyar et al., 2026; Xu et al., 2026). Accordingly, this study defines undergraduates’ AI dependence as a continual and compulsive psychological reliance on AI tools. It is important to distinguish this construct from mere frequent use or functional adoption. Frequent use for academic tasks may reflect adaptive integration of technology into learning, whereas AI dependence, as defined here, refers to compulsive reliance that persists despite negative consequences and progressively displaces independent cognitive effort. Prevalence statistics on frequent AI use should therefore be interpreted as indicators of widespread adoption, while the present study focuses specifically on the mechanisms underlying compulsive dependence. This study adopts the two-dimensional model of functional and emotional dependence proposed by L. Li et al. (2026), which aligns with the definition of psychological reliance and has demonstrated sound psychometric properties. Of note, this model foregrounds functional and emotional facets and does not directly assess cognitive uncritical acceptance of AI-generated content. Cognitive uncritical acceptance, though pedagogically consequential, may involve distinct antecedents and is therefore better treated as a separate construct warranting dedicated investigation. The implications of this measurement choice are addressed in the Discussion and Limitations sections.

The development of AI dependence among undergraduates can be understood as emerging from continuous interactions between environmental influences and individual psychological processes, as conceptualized by social cognitive theory (Bandura, 1989). This framework posits that individual behavior arises from bidirectional influences among individual, behavioral, and contextual factors. Under this framework, the environment not only provides behavioral cues through rules and reinforcement but also is associated with individuals’ cognitive patterns and behavioral habits through peer modeling and social feedback (Wood & Bandura, 1989). Specifically, supportive university climate, as the proximal microenvironment experienced by students, may be linked to their level of reliance on AI tools. This correlation may be partly explained by the transmission of value orientations and behavioral norms concerning technology use (Herman & Lara-Steidel, 2025). Recent studies have identified a close association between supportive university climate and AI dependence. For instance, campus climate perceptions exhibit a positive correlation with academic engagement, whereas reduced engagement tends to be associated with elevated technology dependence (Irshad et al., 2023). Additional empirical findings suggest that perceived teacher support is highly correlated with reduced employment of AI tools among undergraduates (W. Jia et al., 2025). An empirical study involving 553 college students further revealed that perceived faculty support and peer relationships were significantly inversely associated with tendencies toward technology addiction (L. Feng & Zhang, 2022). Although previous studies have preliminarily explored the relationship between supportive university climate and AI dependence among undergraduates, large-scale empirical evidence from mainland China remains scarce. Thus, this study posits the subsequent hypothesis:

**H1.**  *Supportive university climate is negatively associated with undergraduates’ AI dependence.*

### 2.2. The Mediating Role of Academic Self-Efficacy

Academic self-efficacy is defined as students’ confidence in their capacity to attain targeted academic objectives (Schunk & Pajares, 2002) and is closely linked to the effort and persistence they demonstrate when confronting academic challenges. Given the increasing integration of AI into higher education contexts, academic self-efficacy provides a valuable theoretical perspective for understanding students’ interactions with technological tools. A growing body of research has investigated the relationship between academic self-efficacy and AI dependence among undergraduates. Social cognitive theory suggests that uncertainty regarding one’s abilities is often linked to a tendency to seek external support to compensate for perceived deficiencies (Bandura et al., 1999). This perspective has garnered empirical support in the context of AI use. Estrada-Araoz et al. (2025) discovered that academic self-efficacy was significantly related to undergraduates’ AI dependence. L. Zhang and Xu (2025) further reported that undergraduates with higher academic self-efficacy reported less frequent use of AI tools and showed greater learning autonomy. From the perspective of psychological mechanisms, Schutte and Li (2025) believe that students with insufficient academic self-confidence are more likely to be frustrated in the face of challenges, which may be associated with more reliance on AI to obtain instant solutions. Although this short-term coping strategy may temporarily contribute to enhanced performance, it may also be associated with diminished independent thinking and a cycle of technology dependence. Furthermore, studies by Acosta-Enriquez et al. (2025) and S. Zhang et al. (2024) consistently show a negative correlation between academic self-efficacy and AI dependence among undergraduates. This negative association appears consistent across different disciplinary backgrounds and cultural contexts, providing a solid empirical basis for understanding their connection.

As a key environmental factor perceived by students, supportive university climate is closely linked to academic self-efficacy. In accordance with self-efficacy theory, self-efficacy is not a fixed trait but is dynamically associated with interactions with the environment (Q. Wang et al., 2020). In educational settings, contextual factors such as teacher support, peer interactions, and opportunities for self-directed learning can be associated with higher levels of students’ self-efficacy through vicarious experiences and verbal persuasion (Y. Wang et al., 2024). This theoretical proposition has been corroborated by substantial empirical evidence. Farshad et al. (2023) found that positive perceptions of university climate were positively linked to academic self-efficacy among undergraduates. Edman and Brazil (2008), in a study involving 475 college students, further demonstrated that multidimensional perceptions of campus climate (e.g., faculty–student relationships and peer support) were linked to higher levels of academic self-efficacy. Cui et al. (2023) found that the campus learning environment was indirectly linked to students’ self-efficacy through the mediating role of positive learning emotions. L. Zhao et al. (2026) further reported that classroom interaction climate was positively correlated with academic self-efficacy among Chinese undergraduates. Taken together, these studies suggest that supportive university climate is positively associated with academic self-efficacy among undergraduates. Accordingly, this study posits that supportive university climate may be indirectly linked to AI dependence through the mediating role of academic self-efficacy, and proposes the subsequent hypothesis:

**H2.**  *Academic self-efficacy mediates the relationship between supportive university climate and AI dependence among undergraduates.*

### 2.3. The Mediating Role of Self-Control

Self-control refers to individuals’ ability to regulate their emotions, behaviors, and responses in accordance with social expectations or personal standards (Duckworth et al., 2019). In higher education settings, this ability is reflected not only in resistance to distractions (e.g., social media) but also in the maintenance of learning habits that emphasize independent thinking and deep processing, even when immediate answers are readily accessible through AI tools. Accordingly, self-control is regarded as an intrinsic factor associated with lower AI dependence. A growing collection of studies has established an inverse correlation between self-control and AI dependence among undergraduates (Sun et al., 2026). Self-control theory posits that individuals with diminished self-control are more inclined towards quick pleasure and may depend more on external resources for academic task completion, which may be associated with elevated AI dependence (Błachnio et al., 2023). Empirical findings further corroborate this view. Rodríguez-Ruiz et al. (2024), based on a survey of 1761 undergraduates, found that students with lower self-control reported higher levels of adopting AI-generated answers rather than engaging in independent thinking when confronting academic challenges. Similarly, Besalti (2025), in a survey of 449 college students, reported an inverse association between self-control and excessive AI use. Additionally, B. Liu (2026) provided a probable mechanism for this association: self-control is linked to students’ self-regulated learning processes, which are, in turn, associated with their degree of AI dependence.

Furthermore, prior research has explored the relationship between supportive university climate and self-control from a theoretical perspective. According to self-determination theory, when the environment fulfills individuals’ basic psychological needs for autonomy, competence, and relatedness, it may support this internalization process through three pathways: the internalization of external norms and the development of autonomous self-regulation, which in turn may be linked to higher self-control (Darner, 2009). A positive university climate, by meeting students’ basic psychological needs, is considered an important contextual factor linked to students’ self-control (Nakata & Gao, 2025). This proposition is corroborated by empirical evidence. Moilanen et al. (2021), in a survey of college students, found that positive perceptions of university climate were positively linked to self-control. Zhou et al. (2025) further reported that classroom disciplinary climate and peer learning norms are positively associated with students’ self-control. Fang et al. (2025) employed structural equation modeling to find that a supportive campus environment was linked to lower academic anxiety, which in turn was connected to higher self-control. In summary, supportive university climate may be positively associated with undergraduates’ self-control and, through this pathway, indirectly related to lower levels of AI dependence. Therefore, the following hypothesis is proposed:

**H3.**  *Self-control mediates the relationship between supportive university climate and undergraduates’ AI dependence.*

### 2.4. The Sequential Mediation Effect of Academic Self-Efficacy and Self-Control

As indicated by the foregoing literature, academic self-efficacy and self-control may serve important roles in mitigating AI dependence among undergraduates. The directional sequence from academic self-efficacy to self-control is grounded in self-regulation theory: self-efficacy beliefs serve as a motivational foundation that precedes and is theorized to enable self-control (Bandura, 1991). Specifically, students with higher academic self-efficacy anticipate positive outcomes from effort investment and are therefore more motivated to mobilize self-control resources against temptations, including the immediate convenience of AI tools. The belief that “I can do it” is associated with the expectation that sustained effort will be worthwhile, which in turn is linked to self-control deployment being psychologically meaningful. While the reverse sequence, in which self-control enables self-efficacy through sustained effort, is plausible over a longer developmental timescale, the present model concerns the proximal motivational process. A broader reverse causal model also warrants consideration. Drawing on social cognitive theory, mastery experiences are posited to be the primary sources of self-efficacy (Bandura, 1977). Persistent reliance on generative AI may bypass the cognitive struggle required for mastery, which may be associated with progressive decline in academic self-efficacy. This diminished self-efficacy may then be negatively associated with perceptions of the institutional environment, which may correspond to more negative climate appraisals. Both pathways are theoretically grounded and distinguished primarily by temporal scale: the forward model concerns proximal motivational processes, while the reverse model involves a longer-term developmental cycle of cumulative mastery deficits and environmental reinterpretation. The present study examines the forward sequence, with the reverse pathway representing an important hypothesis for future longitudinal research.

From a social cognitive perspective, the environment not only directly relates to behavior but also may indirectly be associated with behavioral choices and persistence through cognitive beliefs and self-regulatory processes (Bandura, 1989). In higher education, supportive university climate, as a key environmental factor, is positively associated with students’ beliefs about their academic capabilities. Undergraduates with elevated academic self-efficacy report stronger self-control when managing distractions and temptations during learning. Self-control, as a key link between cognitive beliefs and actual behavior, may be associated with students’ approaches to tool use in digital learning environments. Students with stronger self-control are more likely to use AI tools strategically, treating them as “cognitive partners” that support learning rather than “cognitive crutches” that replace thinking, thus exhibiting lower levels of AI dependence (X. Chen et al., 2026). Collectively, academic self-efficacy and self-control may function as sequential mediators between supportive university climate and AI dependence, forming an integrated psychological pathway from environmental perception to cognitive belief formation, self-regulatory engagement, and behavioral outcomes. Preliminary empirical evidence offers indirect support for this framework. For example, Alshowkan et al. (2026) found that academic self-efficacy mediates the link between university climate and self-control among undergraduates. Similarly, Demir and Kuşcu Karatepe (2025) reported a positive association between learning self-efficacy and self-control, showing that self-control exhibited an indirect effect in the relationship between learning beliefs and AI dependence.

**Figure 1:**
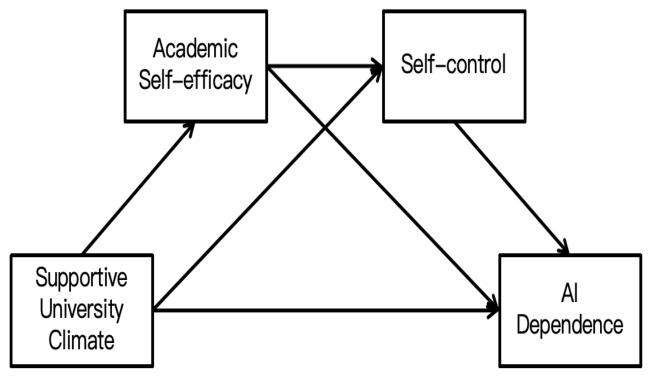
Hypothesized research model.

However, several gaps persist in the existing literature. First, the specific pathways through which academic self-efficacy and self-control connect supportive university climate to undergraduates’ AI dependence have not been systematically examined. Second, limited research has targeted Chinese undergraduates. The mechanisms proposed by social cognitive theory may differ across cultural contexts, and AI dependence among Chinese undergraduates may display context-specific characteristics. Consequently, this research concentrates on Chinese undergraduates. It develops a sequential mediation model based on social cognitive theory (see Figure 1), in which academic self-efficacy and self-control are specified as mediators to investigate the association between supportive university climate and AI dependence. Consequently, the subsequent hypothesis is posited:

**H4.**  *Academic self-efficacy and self-control sequentially mediate the relationship between supportive university climate and AI dependence among undergraduates.*

## 3. Research Methods

### 3.1. Research Sample and Data Sources

This study employed a multi-stage purposive sampling design. Between January and March 2026, participants were recruited from seven full-time undergraduate institutions across four provinces in China, strategically selected to capture diversity in geographic region (eastern, central, and western China) and institutional type (both “Double First-Class” and “Non-Double First-Class” universities). Within each institution, attention was given to securing variation in gender, grade, and major type. Data were collected through a mixed-mode approach combining digital and paper-based modalities. The web-based questionnaire was developed on the Wenjuanxing platform and disseminated via WeChat (version 8.0.66) groups and email with the assistance of university administrators. Offline data were collected in controlled classroom settings, where trained researchers administered paper questionnaires and offered on-site guidance. In total, 1258 surveys were obtained. Following rigorous data screening, invalid responses (e.g., repetitive answers, logical inconsistencies, and excessively short response times) were excluded, yielding 1142 valid questionnaires, with an effective response rate of 90.78%. The demographic characteristics of the sample are presented in Table 1.

### 3.2. Scale Revision and Validation

To improve cultural appropriateness and psychometric reliability for Chinese undergraduates, this study systematically adapted established measurement instruments. Semantic equivalence was initially established through a translation and back-translation procedure, followed by two rounds of content validity evaluation. In the first round, seven experts in higher education psychology assessed item relevance, clarity, and representativeness. In the second round, 12 undergraduates evaluated semantic clarity and situational comprehension, which provides a basis for targeted question revision. In January 2026, a pilot study was conducted with a randomly recruited sample of 156 undergraduates. Reliability analysis revealed that Cronbach’s α coefficients exceeded 0.82 for all dimensions. Exploratory factor analysis indicated adequate sampling adequacy (KMO = 0.872) and a significant Bartlett’s test of sphericity (χ^2^ = 3398.44, *p* < 0.001). According to the standard, the factor load is higher than 0.50, and there is no significant cross-loading. Two items were removed, yielding a distinct four-factor model accounting for 56.17% of the total variance. The factor loadings of the retained items ranged from 0.50 to 0.77, indicating a strong correspondence with the proposed theoretical construct. The final item pool is documented in the Appendix A.

### 3.3. Measurement Instruments

#### 3.3.1. Supportive University Climate Scale

Supportive university climate was measured using an adapted instrument based on the scale originally developed by Y. Jia et al. (2009). The scale comprises 12 items across three dimensions. Responses were captured on a 5-point Likert scale, with higher scores reflecting more positive perceptions of university climate. A sample item is: “Faculty members care about my academic and personal development.” Confirmatory factor analysis (CFA) indicated an adequate model fit (χ^2^/df = 1.852, RMSEA = 0.035, SRMR = 0.018, CFI = 0.973, NFI = 0.958), supporting the scale’s construct validity. The scale demonstrated high internal consistency (Cronbach’s α = 0.921).

#### 3.3.2. Academic Self-Efficacy Scale

Academic self-efficacy was measured using an adapted version of the questionnaire developed by van Zyl et al. (2022). The scale comprises five items. A sample item is: “I believe that as long as I study diligently, I can pass my major course exams.” Responses were recorded on a 5-point Likert scale, where higher scores indicate greater academic self-efficacy. CFA revealed an acceptable model fit (χ^2^/df = 2.186, RMSEA = 0.044, SRMR = 0.036, CFI = 0.912, NFI = 0.903). The scale exhibited adequate internal consistency (Cronbach’s α = 0.875).

#### 3.3.3. Self-Control Scale

Self-control was assessed using the Self-Control Scale (Tangney et al., 2004; Besalti, 2025 revision), comprising 13 items across two dimensions: impulsivity and self-discipline. Higher scores denote greater self-control. A sample item from the impulsivity dimension is: “I can effectively resist the temptation of entertainment and prioritize completing my academic tasks.” Responses were recorded on a 5-point Likert scale, where higher scores denote greater self-control. CFA indicated a satisfactory model fit for the two-factor structure (χ^2^/df = 1.746, RMSEA = 0.032, SRMR = 0.020, CFI = 0.985, NFI = 0.976). The scale demonstrated high internal consistency (Cronbach’s α = 0.918).

#### 3.3.4. AI Dependence Scale

AI dependence was evaluated using a revised measure based on established scales (Y. Chen et al., 2025; W. Li et al., 2026), adapted for the context of Chinese undergraduate education. The scale comprises 12 items across two dimensions. The functional dependence dimension captures difficulty disengaging from AI reliance for academic tasks, while the emotional dimension assesses psychological attachment, such as anxiety when AI is unavailable, together operationalizing compulsive reliance rather than mere frequent use. Sample items include: “I am accustomed to depending on AI tools (e.g., DeepSeek) to complete academic tasks” (functional dependence) and “When unable to access AI, I feel empty or uneasy” (emotional dependence). Responses were recorded on a 5-point Likert scale, with higher scores indicating greater AI dependence. CFA demonstrated an acceptable fit for the two-factor model (χ^2^/df = 2.357, RMSEA = 0.049, SRMR = 0.042, CFI = 0.934, NFI = 0.925). Although the χ^2^/df ratio exceeded the stringent threshold of 2.0 and the CFI and NFI fell below the 0.95 benchmark, the RMSEA and SRMR indicated reasonable approximate fit, and the indices overall support the factorial validity of this adapted scale. The scale showed good internal consistency (Cronbach’s α = 0.880).

### 3.4. Data Analysis

Data analysis was conducted using SPSS and AMOS. First, confirmatory factor analyses were performed in AMOS 26.0 to assess the construct validity of each scale, and Cronbach’s α was calculated to evaluate internal consistency. Second, descriptive statistics and correlation analyses were performed in SPSS 27.0 to characterize the levels of supportive university climate, academic self-efficacy, self-control, and AI dependence, along with their interrelationships. Finally, sequential mediation analyses were conducted using the PROCESS V4. PROCESS was selected for its widespread adoption in mediation research and its ability to generate bias-corrected bootstrap confidence intervals for complex serial mediation models, which facilitates comparison with prior studies. Categorical demographic variables were dummy-coded prior to analysis: Gender (0 = male, 1 = female), Grade (freshman = reference), School type (0 = Non-double first-class, 1 = double first-class), and Major type (natural sciences = reference). After controlling for these demographic covariates, the analyses tested the direct association between supportive university climate and AI dependence, and the independent and sequential mediating roles of academic self-efficacy and self-control.

## 4. Results

### 4.1. Common Method Bias Test

This study used two methods to evaluate common method bias (CMB) (Chin et al., 2012). Harman’s one-factor test revealed that the first factor accounted for 17.911% of the total variance, below the established 40% threshold. To further assess this, the study constructed a confirmatory factor analysis model (M1, four-factor model) and a second model (M2) incorporating latent method factors. The baseline four-factor model (M1) demonstrated adequate fit: χ^2^/df = 2.13, CFI = 0.942, NFI = 0.916, RMSEA = 0.041, SRMR = 0.037. After adding the latent method factor, M2 showed: χ^2^/df = 1.98, CFI = 0.954, NFI = 0.934, RMSEA = 0.028, SRMR = 0.016. The changes in key fit indices were as follows: ΔCFI = 0.012, ΔNFI = 0.018, ΔRMSEA = 0.013, and ΔSRMR = 0.021. The changes in these indices did not surpass the recommended threshold of 0.03, suggesting that adding the common method factor did not significantly improve model fit. These results suggest that adding the common method factor did not substantially improve model fit and that CMB did not significantly distort the factor structure. Nevertheless, all data were self-reported at a single time point, and percept-percept relationships, such as perceived supportive university climate and academic self-efficacy, may be artificially inflated by same-source bias. This should be borne in mind when interpreting these associations.

### 4.2. Descriptive Statistics and Correlation Analysis

Table 2 presents the descriptive statistics and intercorrelations of the four central variables in this study. Descriptive statistics indicate that the means for supportive university climate (M = 3.12, SD = 0.83), academic self-efficacy (M = 3.10, SD = 0.91), and self-control (M = 3.15, SD = 0.81) were at moderate levels, while AI dependence (M = 3.66, SD = 0.56) was comparatively high, approaching the “agree” anchor. This elevated mean should be interpreted with caution, as it may reflect genuine normalization of AI tool use, characteristics of the measurement instrument, or response biases, as discussed below.

Correlation analysis results reveal that supportive university climate is significantly positively correlated with academic self-efficacy (r = 0.562, *p* < 0.01) and self-control (r = 0.528, *p* < 0.01), and significantly negatively correlated with AI dependence (r = −0.402, *p* < 0.01). Additionally, academic self-efficacy was significantly positively correlated with self-control (r = 0.523, *p* < 0.01) and negatively correlated with AI dependence (r = −0.442, *p* < 0.01). At the same time, self-control also showed a significant negative correlation with AI dependence (r = −0.468, *p* < 0.01). These correlations satisfy the prerequisites for testing the hypothesized sequential mediating effects of academic self-efficacy and self-control on the relationship between supportive university climate and AI dependence.

### 4.3. Testing for Mediating Effects

**Figure 2:**
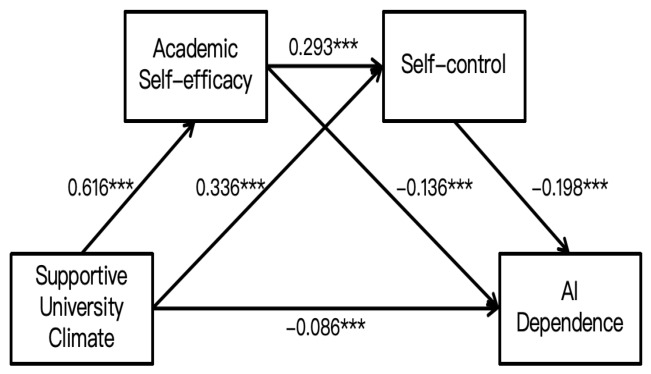
The sequential mediating model (Note: *** indicates *p* < 0.001).

To further investigate the mechanisms underlying supportive university climate and undergraduates’ AI dependence, this study constructed a hypothesized sequential mediation model, with supportive university climate as the independent variable, academic self-efficacy and self-control as mediators, and AI dependence as the outcome. Mediation analysis was performed using PROCESS Model 6 (Hayes, 2012). A bias-corrected bootstrap method was applied with 5000 resamples, and the 95% confidence interval (CI) for the mediation effect was calculated. A mediation effect is deemed significant if the CI does not contain zero. The regression analysis (see Table 3 and Figure 2) reveals that the model accounted for 16.3% of the variance in AI dependence (R^2^ = 0.163, F = 44.189, *p* < 0.001), reflecting an acceptable level of explained variance. Supportive university climate was significantly positively associated with academic self-efficacy (β = 0.616, t = 22.93, *p* < 0.001) and self-control (β = 0.336, t = 11.94, *p* < 0.001), and negatively associated with AI dependence (β = −0.086, t = −3.93, *p* < 0.001), which is consistent with Hypothesis 1. Academic self-efficacy was significantly positively correlated with self-control (β = 0.293, t = 11.40, *p* < 0.001) and negatively correlated with AI dependence (β = −0.136, t = −6.87, *p* < 0.001). Self-control was also significantly negatively correlated with AI dependence (β = −0.198, t = −9.12, *p* < 0.001).

The sequential mediation analysis results (see Table 4) indicate that the direct effect of supportive university climate on AI dependence is −0.086, with a total indirect effect of −0.186, rep-resenting 31.62% and 68.38% of the total effect, respectively. The three mediation pathways are described below:

(1) The indirect effect of supportive university climate → academic self-efficacy → AI dependence was −0.084 (Boot CI = [−0.11, −0.06]), accounting for 30.88% of the total indirect effect. As the CI does not include zero, the mediating effect of academic self-efficacy is significant, supporting Hypothesis 2.
(2) The indirect effect of supportive university climate → self-control → AI dependence was −0.067 (Boot CI = [−0.08, −0.05]), making up 24.63% of the total indirect effect. The CI excludes zero, indicating a significant mediating effect of self-control, consistent with Hypothesis 3.
(3) The sequential mediation effect of supportive university climate → academic self-efficacy → self-control → AI dependence was −0.036 (Boot CI = [−0.05, −0.03]), representing 13.24% of the total indirect effect. The CI excludes zero, indicating that the sequential mediation effect is significant, supporting Hypothesis 4.

## 5. Discussion

### 5.1. Supportive University Climate and Undergraduates’ AI Dependence

This study reveals a significant negative correlation between supportive university climate and AI dependence. This finding extends prior research, which has focused primarily on individual-level antecedents, by demonstrating that a supportive campus climate is also associated with undergraduates’ AI dependence. According to ecological systems theory, supportive university climate, as the microsystem most directly perceived by students, is associated with their psychological orientation toward AI tools by conveying implicit messages about learning values, technology use norms, and social expectations (Bronfenbrenner, 1977). Mechanistically, the three supportive dimensions examined may operate through distinct yet complementary pathways: teacher support through social persuasion, peer support through vicarious experiences, and autonomy through self-directed exploration (Bandura et al., 1999). Existing research indirectly corroborates this inference. For instance, Huang and Wang (2023) found that students perceiving higher faculty support and autonomy opportunities exhibited greater intrinsic motivation and lower academic burnout. Coetzee et al. (2022) reported that a supportive university climate is inversely correlated with the tendency for misuse in instrumental learning behaviors. An alternative interpretation should be noted: because all constructs were self-reported, baseline psychological states such as anxiety, depression, or academic stress could simultaneously shape perceptions of supportive university climate and influence the mediating and outcome variables. The supportive university climate measure, however, captures specific institutional features that are conceptually distinct from general affective states, and previous studies have reported associations between institutional climate and student outcomes that remain significant after accounting for individual differences in affective states.

The relatively high mean score for AI dependence may also reflect several factors. First, AI tools have become pervasively integrated into undergraduates’ academic routines, and a certain level of instrumental reliance may have become a socially accepted norm. Second, the measurement instrument, which captures functional and emotional facets of dependence, may tap into both adaptive habitual use and problematic psychological reliance. In the absence of validated cut-off scores, categorically distinguishing adaptive from pathological dependence remains challenging, and the interpretation of what constitutes “dependence” therefore relies on the content validity of the instrument. Third, social desirability is unlikely to inflate such scores, but acquiescence bias cannot be entirely ruled out. Notably, the present findings pertain solely to the supportive facets of university climate. Whether competitive or pressure-oriented dimensions exhibit distinct patterns of association with AI dependence remains an open empirical question. Therefore, fostering a supportive university climate carries practical implications for preventing excessive AI dependence among students.

### 5.2. The Mediating Role of Academic Self-Efficacy

This study demonstrates that academic self-efficacy mediates the link between supportive university climate and AI dependence among undergraduates. Social cognitive theory suggests that this process should be understood from a dynamic perspective, emphasizing continuous interaction between individuals and their environment. Supportive university climate interacts with student needs, which may be directly linked to AI dependence or indirectly connected to it through academic self-efficacy. The results align with the hypothesized pathway: “supportive university climate → academic self-efficacy → AI dependence.” First, a positive university climate is significantly correlated with higher academic self-efficacy among undergraduates, consistent with previous studies (Shi & Ko, 2023). In Chinese undergraduate education, academic self-efficacy is primarily associated with direct experience, vicarious learning, social persuasion, and emotional arousal (Usher & Pajares, 2008). For instance, when students receive timely feedback and encouragement from instructors or observe peers earning awards through their own efforts, these experiences may be associated with their positive self-assessment; simultaneously, positive emotions are linked to intrinsic beliefs in their own efficacy. Moreover, the rich learning resources, opportunities for independent exploration, and tolerance for innovative thinking offered by universities are associated with greater chances for students to succeed. Second, academic self-efficacy among undergraduates is significantly negatively correlated with AI dependence. Undergraduates with higher academic self-efficacy are inclined to view academic challenges as opportunities to be mastered through their own efforts, prioritizing cognitive resource mobilization over immediate reliance on AI tools. Particularly in the context of Chinese undergraduate education, which emphasizes “a solid foundation and strong capabilities,” strong academic self-efficacy is associated with positive achievement expectations grounded in self-awareness, which may be related to maintained agency when using AI (Pan, 2020). This orientation may help students avoid emotional dependence stemming from low confidence, and is associated with stronger academic resilience and greater depth of independent thinking. The relatively larger indirect effect observed for this pathway (30.88% of the total indirect effect) underscores the central role of academic self-efficacy in the association between environmental perceptions and technology-related behavioral outcomes.

### 5.3. The Mediating Role of Self-Control

The research demonstrates that self-control mediates the relationship between supportive university climate and AI dependence among undergraduates. Specifically, a supportive university climate is inversely correlated with AI dependence both directly and indirectly through its positive relationship with self-control. This is consistent with the notion that multiple pathways may connect supportive university climate to AI dependence through the behaviors they elicit. Previous studies have documented a significant positive correlation between supportive university climate and self-control (Liang et al., 2026). In Chinese universities, a supportive climate—characterized by faculty support, peer collaboration, and opportunities for autonomous decision-making—is positively linked to the development and strengthening of students’ self-control abilities (C. Li, 2025; Y.-D. Yang et al., 2024). Self-control is also significantly inversely correlated with undergraduates’ overreliance on AI. Existing research indicates that self-control, a key capacity to inhibit impulses and pursue long-term goals, is inversely correlated with problematic technology use (Fan et al., 2022). Scholars suggest that individuals with strong self-control are more likely to weigh long-term academic goals against short-term gains when confronted with the immediate convenience of AI, which may be related to greater resistance to the impulse to depend on it (X. Zhao et al., 2025). Therefore, greater self-control among undergraduates is associated with lower levels of AI dependence. The present findings are consistent with the limited resources theory of self-control. This theory posits that self-control is a finite and depletable resource (Muraven & Baumeister, 2000). A supportive university environment, which provides faculty and peer support along with opportunities for autonomous decision-making, may be associated with lower levels of resource depletion during self-control, which in turn may be related to more effective resistance to excessive use of AI. Conversely, without such supportive elements, students face increased academic competition and the “shortcuts” offered by AI. This may be associated with continuous self-control exertion and resource depletion. When self-control resources are low, students may find it more difficult to regulate their technology use and may be more likely to exhibit AI dependence. Based on this analysis, universities should consider the rational allocation and replenishment of students’ self-control resources to prevent excessive AI dependence, potentially resulting from resource depletion. The substantial indirect effect through self-control (24.63% of the total indirect effect), though slightly smaller than that through self-efficacy, highlights the independent contribution of self-regulatory capacity in mitigating AI dependence.

### 5.4. The Sequential Mediation Effect of Academic Self-Efficacy and Self-Control

The findings support a sequential mediation pathway in which supportive university climate is positively associated with academic self-efficacy, which in turn is linked to stronger self-control and, ultimately, lower AI dependence. Resource conservation theory offers an explanatory lens: institutional support is associated with self-efficacy as a psychological resource, which is linked to the deployment of self-control as a behavioral resource, which may help students to resist AI shortcuts and avert skill atrophy (Hobfoll, 1989; Meier et al., 2016). Thus, academic self-efficacy and self-control form a resource transmission chain linking supportive university climate to lower AI dependence. A reverse causal model is also theoretically grounded. Drawing on social cognitive theory, mastery experiences are posited to be the primary sources of self-efficacy (Bandura, 1977). Persistent AI reliance may bypass the cognitive struggle necessary for mastery, being associated with progressive decline in academic self-efficacy. Diminished self-efficacy may then be associated with more negative perceptions of the institutional environment, which may correspond to more negative climate appraisals. The present cross-sectional design cannot adjudicate between these pathways; they may operate reciprocally over time, forming a feedback loop that warrants longitudinal investigation. The significant residual direct effect (−0.086, 31.62% of the total effect) indicates that cognitive, emotional, and social-normative factors not assessed in this study may further account for the climate–dependence association. Regarding the relative importance of the three indirect pathways, academic self-efficacy carried the largest effect (30.88%), followed by self-control (24.63%) and the sequential chain (13.24%). This pattern suggests that self-efficacy serves as the primary psychological filter through which institutional resources are internalized, while self-control, partly dependent on self-efficacy, provides a complementary pathway.

### 5.5. Theoretical Implications

This study makes the following theoretical contribution. First, this study identifies a negative correlation between supportive university climate and AI dependence among undergraduates, which suggests that supportive university climate may function as an external environmental factor associated with AI dependence among undergraduates. This finding offers novel empirical evidence for understanding the factors that are correlated with undergraduates’ AI dependence. Second, by specifying academic self-efficacy and self-control as sequential mediators, this study extends social cognitive theory by identifying a directional “belief-driven regulation” pathway and enriches resource conservation theory (Hobfoll, 1989) by illustrating a resource transmission chain from institutional support to psychological and behavioral resources. The distinct indirect pathways further reveal that self-efficacy and self-control function both independently and sequentially, underscoring their complementary roles in the association between the environment and technology-related behavior. Finally, using a sample of Chinese undergraduates, this study examines AI dependence within the context of China’s higher education system, thus contributing to cross-cultural discourse in this field. The findings highlight the cultural specificity of the relationship between supportive university climate and undergraduates’ AI dependence in a global context, providing empirical evidence from China to inform the development of a locally relevant technology-psychological theory. It should be noted that the AI dependence measure used in this study focuses on functional and emotional facets of reliance and does not directly assess cognitive dependence (i.e., uncritical acceptance of AI-generated content). The theoretical pathways identified here are therefore empirically grounded in these two facets; their generalizability to cognitive dependence awaits further investigation.

### 5.6. Practical Implications

This study provides practical implications for higher education institutions seeking to implement systematic prevention and intervention strategies for AI dependence among undergraduates. First, universities should consider enhancing campus climate across multiple dimensions to cultivate an environment that supports rational AI use among students. Regarding faculty support, universities should encourage instructors to emphasize the value of deep learning during classroom interactions and academic mentoring, which may help students recognize AI as an auxiliary tool. Concerning peer support, universities can establish collaborative learning communities to encourage an atmosphere of peer support and collective resistance to technology misuse, which may encourage students in their internalization of norms of responsible AI use through daily interactions. Regarding opportunities for autonomy, universities can delegate authority in course assessments and student management to support students’ perceived control over their academic behaviors and strengthen their intrinsic motivation (Shukla & Arora, 2023).

Second, universities should focus on enhancing undergraduates’ academic self-efficacy as a means to guide students to develop a rational understanding of AI. Specifically, universities can employ tiered learning tasks to provide opportunities to build efficacy through successful experiences, use peer experience-sharing to provide alternative role models, and offer timely, specific positive feedback to support students’ perception of their own capabilities. With this foundation, course design should underscore the importance of critical thinking when using AI, which may help students appreciate the irreplaceable value of their cognitive efforts (Ma et al., 2025). When academic self-efficacy is higher, students may be more likely to perceive AI as a “tool” for expanding their capabilities rather than a “shortcut” for avoiding thinking (Estrada-Araoz et al., 2025).

Third, universities should prioritize developing students’ self-control skills to strengthen internal safeguards for responsible AI use. Through specialized workshops or structured instruction, universities can systematically teach self-regulation strategies, such as goal-setting and impulse management, which may assist students in establishing personal guidelines for AI use and in determining the contexts and frequency of usage (Husna et al., 2024). Additionally, universities should support students in promptly recognizing and correcting tendencies toward overdependence, which may contribute to cultivating the ability to exercise rational control over technology use.

## 6. Limitations and Future Directions

Several limitations should be acknowledged. First, the cross-sectional design limits causal inference. All data were self-reported at a single time point, constraining temporal precedence and introducing CMB. Although statistical tests suggested that CMB did not substantially distort the factor structure, percept-percept relationships may remain inflated. The mediation models should therefore be interpreted as testing plausible theoretical pathways consistent with the data, not as establishing causal sequences. Two alternative directional models cannot be ruled out: the reverse sequence in which self-control enables self-efficacy through sustained effort, and the broader reverse pathway in which persistent AI dependence erodes self-efficacy by bypassing mastery experiences and negatively biases climate perceptions. Baseline psychological states such as anxiety, depression, and academic stress were not assessed and may confound the observed associations. Future research should adopt longitudinal or experimental designs with cross-lagged panel models, incorporate psychological well-being measures as controls, and employ multi-informant designs with objective behavioral indicators to mitigate CMB.

Second, both key constructs face measurement limitations. The supportive university climate scale captured only supportive facets. The AI dependence scale assessed functional and emotional dependence but omitted cognitive uncritical acceptance. Additionally, the elevated mean and absence of validated cut-offs limit categorical distinctions between adaptive and problematic dependence, and self-report measures may not fully differentiate the two. Future research should incorporate competitive climate measures, employ multidimensional AI dependence instruments integrating cognitive, behavioral, and emotional facets, and develop empirically grounded thresholds to distinguish adaptive from problematic use.

Third, the sample was drawn from seven universities across four provinces in China. Although regional and institutional diversity was sought, recruitment relied on institutional contacts and convenience-based classroom access rather than probability sampling, limiting representativeness. Future research should adopt rigorous probability sampling with transparent documentation and conduct cross-cultural comparisons to test external validity.

Fourth, methodological constraints exist. Mediation analyses used the PROCESS macro, which relies on observed composite scores and does not model latent measurement error. Future research should test the full structural equation model with latent variables. The CFA fit indices for the AI dependence scale were acceptable but less strong than those for other measures, suggesting further psychometric refinement is warranted.

Finally, the significant residual direct effect indicates that additional unmeasured mediators may operate alongside self-efficacy and self-control, including critical thinking disposition, AI literacy, academic anxiety, and peer AI use norms (Kong et al., 2025; Yue et al., 2020; H. Zhang & Pan, 2026). Future research incorporating these specific variables into comprehensive models is recommended. Integrating qualitative methods could further illuminate the contexts and motivations underlying students’ AI use, advancing a context-sensitive theoretical framework.

## 7. Conclusions

The research investigates the correlation between supportive university climate and undergraduates’ AI dependence in the Chinese educational system, emphasizing the sequential mediating effects of academic self-efficacy and self-control. The primary conclusions are as follows: (1) supportive university climate is negatively associated with undergraduates’ AI dependence; (2) academic self-efficacy serves as a mediator connecting supportive university climate to AI dependence among undergraduates; (3) self-control mediates the relationship between supportive university climate and undergraduates’ AI dependence; (4) supportive university climate and undergraduates’ AI dependence are sequentially mediated by academic self-efficacy and self-control. Based on these findings, universities can design educational interventions to support students’ rational AI use through a supportive campus climate and coordinated “cognitive–behavioral” strategies. Specifically, institutions should refine academic assessment and support systems to support students’ academic self-efficacy, while incorporating self-control strategies and responsible digital tool use education, with emphasis on cultivating students’ ability to self-regulate their technology use. These measures may support students in developing discerning habits in using AI, potentially contributing to a foundation for their sustainable development in an AI-driven academic and personal growth environment.

## Figures and Tables

**Table 1:** Sample demographics (n = 1142).

Variable	Classification	Number (n)	Percentage (%)
Gender	Male	544	47.64%
	Female	598	52.36%
Grade	Freshman	268	23.47%
	Sophomore	318	27.85%
	Junior	291	25.48%
	Senior	265	23.20%
School type	Non-double first-class	602	52.71%
	Double first-class	540	47.29%
Major type	Natural sciences	408	35.73%
	Humanities	377	33.01%
	Social sciences	357	31.26%

**Table 2:** Descriptive statistics and correlation coefficients (n = 1142).

Variable	M	SD	1	2	3	4
1. Supportive university climate	3.12	0.83	1			
2. Academic self-efficacy	3.10	0.91	0.562 **	1		
3. Self-control	3.15	0.81	0.528 **	0.523 **	1	
4. AI dependence	3.66	0.56	−0.402 **	−0.442 **	−0.468 **	1

Note: ** indicates *p* < 0.01.

**Table 3:** Mediating analysis results (n = 1142).

Outcome Variable	Predictor Variables	R^2^	F	β	SE	t	*p*
Academic self-efficacy	Constant	0.319	106.430 ***	0.966	0.15	6.47	0.00
	Gender			0.015	0.04	0.32	0.75
	Grade			0.020	0.02	0.98	0.33
	School type			0.031	0.04	0.68	0.50
	Major type			0.047	0.03	1.72	0.09
	Supportive university climate			0.616 ***	0.03	22.93	0.00
Self-control	Constant	0.356	104.666 ***	1.058	0.13	8.04	0.00
	Gender			−0.006	0.04	−0.16	0.87
	Grade			0.028	0.02	1.55	0.12
	School type			0.056	0.04	1.44	0.15
	Major type			0.001	0.02	0.02	0.99
	Supportive university climate			0.336 ***	0.03	11.94	0.00
	Academic self-efficacy			0.293 ***	0.03	11.40	0.00
AI dependence	Constant	0.283	63.907 ***	4.959	0.10	50.07	0.00
	Gender			−0.012	0.03	−0.44	0.66
	Grade			−0.002	0.01	−0.16	0.88
	School type			0.030	0.03	1.06	0.29
	Major type			−0.002	0.02	−0.12	0.91
	Supportive university climate			−0.086 ***	0.02	−3.93	0.00
	Academic self-efficacy			−0.136 ***	0.02	−6.87	0.00
	Self-control			−0.198 ***	0.02	−9.12	0.00

Note: *** indicates *p* < 0.001.

**Table 4:** Path analysis results (n = 1142).

	Effect	Boot SE	Boot LLCI	Boot ULCI	Effect Rate
Total effect	−0.272	0.02	−0.31	−0.24	100%
Direct effect	−0.086	0.02	−0.13	−0.04	31.62%
Total mediation effect	−0.186	0.02	−0.22	−0.15	68.38%
Supportive university climate → Academic self-efficacy → AI dependence	−0.084	0.01	−0.11	−0.06	30.88%
Supportive university climate → Self-control → AI dependence	−0.067	0.01	−0.08	−0.05	24.63%
Supportive university climate → Academic self-efficacy → Self-control → AI dependence	−0.036	0.01	−0.05	−0.03	13.24%

## Data Availability

Data from this study can be accessed from the corresponding author upon reasonable request.

## Appendix A

**Supportive university climate:**
  1. Faculty members care about my academic and personal development.
  2. I can receive effective guidance from faculty when encountering academic difficulties.
  3. Faculty members attend to and affirm my academic progress.
  4. Faculty encouragement strengthens my academic confidence.
  5. I have opportunities to participate in discussions about classroom rules or instructional arrangements.
  6. Faculty members consider students’ interests and needs in their teaching.
  7. Students have room for autonomous decision-making in course tasks or activities.
  8. I can express my views on class or course-related matters.
  9. Students respect each other and maintain harmonious relationships.
  10. I can receive academic or personal help from classmates when needed.
  11. I rarely experience malicious conflict or exclusion among classmates around me.
  12. I trust my classmates and am willing to collaborate with them on academic tasks.

**Academic self-efficacy:**
  1. I believe that as long as I study diligently, I can pass my major course exams.
  2. I can persist in and achieve my academic goals in my major.
  3. I can remain calm during exams because I believe I can answer the questions well.
  4. If I work hard enough, I can generally solve challenging problems in my major courses.
  5. In my studies, I feel that “if others can do it, I can too.”

**Self-control:**
  1. I can effectively resist entertainment temptations and prioritize completing academic tasks. (R)
  2. I can proactively overcome bad habits such as procrastination and staying up late. (R)
  3. I can control myself from overindulging in entertaining but unproductive activities. (R)
  4. I can firmly refuse things that are detrimental to my studies or health. (R)
  5. When I realize my behavior is inappropriate, I can stop promptly. (R)
  6. Before making important decisions, I fully consider the pros and cons of various options. (R)
  7. I can maintain a diligent and focused state in daily study and life.
  8. I can speak and act prudently in class or group discussions.
  9. I am satisfied with my current self-discipline ability.
  10. In the eyes of my classmates, I am a highly self-disciplined person.
  11. I can manage my time effectively and avoid letting entertainment interfere with important matters.
  12. I can maintain concentration for extended periods while studying.
  13. I can make plans for long-term goals and execute them effectively.

**AI dependence:**
  1. I am accustomed to depending on AI tools (e.g., DeepSeek) to complete academic tasks.
  2. When encountering academic problems, I turn to AI first rather than engaging in deep thinking on my own.
  3. I often rely on AI suggestions to make academic or daily decisions.
  4. Without access to AI, completing certain academic tasks becomes difficult for me.
  5. I often spend more time using AI than originally planned.
  6. After frequent AI use, I feel that my willingness or ability to think independently has declined.
  7. I use AI to relieve negative emotions.
  8. When feeling lonely, I turn to AI for companionship.
  9. When communicating with AI, I feel understood.
  10. I confide in AI about personal matters I am unwilling to share with others.
  11. When unable to access AI, I feel empty or uneasy.
  12. Interacting with AI has become one of my daily relaxation habits.

## References

[B1-behavsci-16-00902] Acosta-Enriquez B. G., Ballesteros M. A. A., Guzman Valle M. d. l. A., Angaspilco J. E. M., Aquino Lalupú J. d. R., Jaico J. L. B., Germán Reyes N. C., Alarcón García R. E., Castillo W. E. J. (2025). The mediating role of academic stress, critical thinking and performance expectations in the influence of academic self-efficacy on AI dependence: Case study in college students. Computers and Education: Artificial Intelligence.

[B2-behavsci-16-00902] Al Mashagbeh M., Alsharqawi M., Tudevdagva U., Khasawneh H. J. (2025). Student engagement with artificial intelligence tools in academia: A survey of Jordanian universities. Frontiers in Education.

[B3-behavsci-16-00902] Almassaad A., Alajlan H., Alebaikan R. (2024). Student perceptions of generative artificial intelligence: Investigating utilization, benefits, and challenges in higher education. Systems.

[B4-behavsci-16-00902] Alshowkan A., Shdaifat E., Alnass F., Al-Dhafeeri F. (2026). Academic atmosphere, academic self-perception, and student self-efficacy: A structural model. BMC Nursing.

[B5-behavsci-16-00902] Anderson C. S. (1982). The search for school climate: A review of the research. Review of Educational Research.

[B6-behavsci-16-00902] Arowosegbe A., Alqahtani J. S., Oyelade T. (2024). Perception of generative AI use in UK higher education. Frontiers in Education.

[B7-behavsci-16-00902] Bandura A. (1977). Self-efficacy: Toward a unifying theory of behavioral change. Psychological Review.

[B8-behavsci-16-00902] Bandura A. (1989). Human agency in social cognitive theory. American Psychologist.

[B9-behavsci-16-00902] Bandura A. (1991). Social cognitive theory of self-regulation. Organizational Behavior and Human Decision Processes.

[B10-behavsci-16-00902] Bandura A., Freeman W. H., Lightsey R. (1999). Self-efficacy: The exercise of control. Journal of Cognitive Psychotherapy.

[B11-behavsci-16-00902] Berhanu K. Z., Sewagegn A. A. (2024). The role of perceived campus climate in students’ academic achievements as mediated by students’ engagement in higher education institutions. Cogent Education.

[B12-behavsci-16-00902] Besalti M. (2025). Harnessing self-control and AI: Understanding ChatGPT’s impact on academic wellbeing. Behavioral Sciences.

[B13-behavsci-16-00902] Błachnio A., Przepiorka A., Cudo A., Angeluci A., Ben-Ezra M., Durak M., Kaniasty K., Mazzoni E., Senol-Durak E., Hou W. K., Benvenuti M. (2023). Self-control and digital media addiction: The mediating role of media multitasking and time style. Psychology Research and Behavior Management.

[B14-behavsci-16-00902] Bronfenbrenner U. (1977). Toward an experimental ecology of human development. American Psychologist.

[B15-behavsci-16-00902] Chan C. K. Y., Hu W. (2023). Students’ voices on generative AI: Perceptions, benefits, and challenges in higher education. International Journal of Educational Technology in Higher Education.

[B16-behavsci-16-00902] Chen X., Cui M., Sun P., Xu X., Ma K., Yan L. (2026). Unpacking the relationship between self-control and academic engagement: The mediating role of meaning in life and the moderating role of GenAI dependence. BMC Psychology.

[B17-behavsci-16-00902] Chen Y., Wang M., Yuan S., Zhao Y. (2025). Development and validation of the conversational AI dependence scale for Chinese college students. Frontiers in Psychology.

[B18-behavsci-16-00902] Chin W. W., Thatcher J. B., Wright R. T. (2012). Assessing common method bias: Problems with the ULMC technique. MIS Quarterly.

[B19-behavsci-16-00902] Coetzee T., Pryce-Jones K., Grant L., Tindle R. (2022). Hope moderates the relationship between students’ sense of belonging and academic misconduct. International Journal for Educational Integrity.

[B20-behavsci-16-00902] Cohen J., McCabe E. M., Michelli N. M., Pickeral T. (2009). School climate: Research, policy, practice, and teacher education. Teachers College Record.

[B21-behavsci-16-00902] Cui Y., Liu Z., Sun Z., Jin J. (2023). Campus learning environment, the influence of psychological emotions during learning on self-learning efficacy. Proceedings of the 2023 8th international conference on distance education and learning.

[B22-behavsci-16-00902] Darner R. (2009). Self-determination theory as a guide to fostering environmental motivation. The Journal of Environmental Education.

[B23-behavsci-16-00902] Demir S., Kuşcu Karatepe H. (2025). The effect of academic procrastination on life satisfaction among nursing and midwifery students: The serial mediation role of academic self-efficacy and self-control. Behavioral Sciences.

[B24-behavsci-16-00902] Duckworth A. L., Taxer J. L., Eskreis-Winkler L., Galla B. M., Gross J. J. (2019). Self-control and academic achievement. Annual Review of Psychology.

[B25-behavsci-16-00902] Edman J. L., Brazil B. (2008). Perceptions of campus climate, academic efficacy and academic success among community college students: An ethnic comparison. Social Psychology of Education.

[B26-behavsci-16-00902] Estrada-Araoz E. G., Mamani-Roque M., Quispe-Aquise J., Manrique-Jaramillo Y. V., Cruz-Laricano E. O. (2025). Academic self-efficacy and dependence on artificial intelligence in a sample of university students. Sapienza: International Journal of Interdisciplinary Studies.

[B27-behavsci-16-00902] Fan Z., Chen M., Lin Y. (2022). Self-control and problematic internet use in college students: The chain mediating effect of rejection sensitivity and loneliness. Psychology Research and Behavior Management.

[B28-behavsci-16-00902] Fang Z., Fu Y., Liu D., Chen C. (2025). The impact of school climate on college students’ socio-emotional competence: The mediating role of psychological resilience and emotion regulation. BMC Psychology.

[B29-behavsci-16-00902] Farshad F., Kheirkhah M., Virtanen J., Hessari H. (2023). Role of the educational atmosphere on self-efficacy among dental students. Strides in Development of Medical Education.

[B30-behavsci-16-00902] Feng L., Zhang L. (2022). Perceived teacher support, peer relationship, and university students’ mental health: The mediation of reality and Internet altruistic behaviors. Frontiers in Psychology.

[B31-behavsci-16-00902] Feng S., Zhou H. (2025). Heterogeneous patterns of generative AI use among Chinese university students: Application depth, benefits, and dependency. Exploring Education Development.

[B32-behavsci-16-00902] Fieldhouse R. (2025). AI is saving time and money in research—But at what cost?. Nature.

[B33-behavsci-16-00902] Gerlich M. (2025). AI tools in society: Impacts on cognitive offloading and the future of critical thinking. Societies.

[B34-behavsci-16-00902] Hayes A. F. (2012). PROCESS: A versatile computational tool for observed variable mediation, moderation, and conditional process modeling.

[B35-behavsci-16-00902] Herman J., Lara-Steidel H. (2025). Artificial intelligence on campus: Revisiting understanding as an aim of higher education. Educational Theory.

[B36-behavsci-16-00902] Hobfoll S. E. (1989). Conservation of resources: A new attempt at conceptualizing stress. American Psychologist.

[B37-behavsci-16-00902] Hoy W. K. (1990). Organizational climate and culture: A conceptual analysis of the school workplace. Journal of Educational and Psychological Consultation.

[B38-behavsci-16-00902] Huang L., Wang D. (2023). Teacher support, academic self-efficacy, student engagement, and academic achievement in emergency online learning. Behavioral Sciences.

[B39-behavsci-16-00902] Husna R., Ramadhani S., A’yun Q. (2024). The role of self-control in overcoming ethical challenges in the development of artificial intelligence. BICC Proceedings.

[B40-behavsci-16-00902] Irshad M., Qureshi M. A., Saraih U. N., Ahmad S. F. (2023). Impact of institutional climate on the student’s engagement and learning outcomes in private sector universities of Karachi. International Journal of Management in Education.

[B41-behavsci-16-00902] Jia W., Pan L., Neary S. (2025). Effect of GenAI dependency on university students’ academic achievement: The mediating role of self-efficacy and moderating role of perceived teacher caring. Behavioral Sciences.

[B42-behavsci-16-00902] Jia Y., Way N., Ling G., Yoshikawa H., Chen X., Hughes D., Ke X., Lu Z. (2009). The influence of student perceptions of school climate on socioemotional and academic adjustment: A comparison of Chinese and American adolescents. Child Development.

[B43-behavsci-16-00902] Kang X., Liu M., Wu S. (2025). How the university campus climate promotes the development of social-emotional competence in pre-service teachers: A mediation of professional identity and moderation of relative deprivation?. Journal of Educational Science of Hunan Normal University.

[B44-behavsci-16-00902] Kong L., Zhao M., Huang W., Zhang W., Liu J. (2025). The impact of academic anxiety on smartphone addiction among college students: The mediating role of self-regulatory fatigue and the moderating role of mindfulness. BMC Psychology.

[B45-behavsci-16-00902] Li C. (2025). Do teacher-training college students become more engaged in their studies because of commitment? The mediating role of self-control and the moderating role of core self-evaluation. Frontiers in Psychology.

[B46-behavsci-16-00902] Li L., Wang T., Wang Y., Wang Y., Zeng X. (2026). Development and validation of the conversational AI dependence scale (CAID): A dual-dimensional measure of instrumental and emotional dependence. International Journal of Human Computer Interaction.

[B47-behavsci-16-00902] Li W., Lu J., Cao L., Xiao M. (2026). University organizational support and engineering identity among engineering post-graduates: The sequential mediating role of major satisfaction and engineering belonging. Behavioral Sciences.

[B48-behavsci-16-00902] Liang J., Yin X., Wei X., Yang X., Ji Y. (2026). Perceived stress and mobile phone addiction among Chinese undergraduate nursing students: The mediating role of organizational caring climate and self-control. Frontiers in Psychology.

[B49-behavsci-16-00902] Liu B. (2026). How does self-regulated learning resist AI dependence? A mediating effect study based on college students who frequently use AIGC tools. Sage Open.

[B50-behavsci-16-00902] Liu X., Liu Y., Dai Y., Fu J. (2026). Academic stress and university students’ dependency on generative artificial intelligence: A multiple mediation model using PLS-SEM. BMC Psychology.

[B51-behavsci-16-00902] Ma Y., Su Y., Li M., Zhang Y., Chai W., Huang A., Zhao X. (2025). Preparing students for an AI-driven world: Generative AI and curriculum reform in higher education. Frontiers of Digital Education.

[B52-behavsci-16-00902] Meier A., Reinecke L., Meltzer C. E. (2016). “Facebocrastination”? Predictors of using Facebook for procrastination and its effects on students’ well-being. Computers in Human Behavior.

[B53-behavsci-16-00902] Moilanen K. L., DeLong K. L., Spears S. K., Gentzler A. L., Turiano N. A. (2021). Predictors of initial status and change in self-control during the college transition. Journal of Applied Developmental Psychology.

[B54-behavsci-16-00902] Muraven M., Baumeister R. F. (2000). Self-regulation and depletion of limited resources: Does self-control resemble a muscle?. Psychological Bulletin.

[B55-behavsci-16-00902] Nakata Y., Gao X. (2025). Why classroom climate matters: Exploring Japanese university students’ motivational regulation within a classroom ecology. Language Teaching Research.

[B56-behavsci-16-00902] Nilsen F. A., Bang H., Boe O., Martinsen Ø. L., Lang-Ree O. C., Røysamb E. (2020). The multidimensional self-control scale (MSCS): Development and validation. Psychological Assessment.

[B57-behavsci-16-00902] OECD (2026). OECD digital education outlook 2026: Exploring effective uses of generative AI in education.

[B58-behavsci-16-00902] Pamuk M. (2026). Investigation of the serial mediation roles of social media craving and self-control in the relationship between social media addiction and psychological adjustment problems of university students. Current Psychology.

[B59-behavsci-16-00902] Pan X. (2020). Technology acceptance, technological self-efficacy, and attitude toward technology-based self-directed learning: Learning motivation as a mediator. Frontiers in Psychology.

[B60-behavsci-16-00902] Rodríguez-Ruiz J., Marín-López I., Espejo-Siles R. (2024). Is artificial intelligence use related to self-control, self-esteem and self-efficacy among university students?. Education and Information Technologies.

[B61-behavsci-16-00902] Schunk D. H., Pajares F., Wigfield A., Eccles J. S. (2002). The development of academic self-efficacy. Development of achievement motivation.

[B62-behavsci-16-00902] Schutte N. S., Li H. (2025). The role of self-efficacy and curiosity in student use of artificial intelligence (AI). International Journal of Educational Technology in Higher Education.

[B63-behavsci-16-00902] Shi Y., Hui X., Li G., Ouyang M., Yang C. (2025). Boredom proneness and Chinese vocational college students’ academic disengagement: Mediating role of academic self-efficacy and moderating role of self-control. Frontiers in Psychology.

[B64-behavsci-16-00902] Shi Y., Ko Y. C. (2023). A study on the influence of family and school psychological environment on academic self-efficacy and self-identity of English education major university students. Participatory Educational Research.

[B65-behavsci-16-00902] Shukla A., Arora V. (2023). A holistic approach to student empowerment and assessment of its impact on educational outcomes through psychological ownership. Studies in Higher Education.

[B66-behavsci-16-00902] Singer-Freeman K. E., Verbeke K., Barre B. (2025). Generative AI use among university students depends on academic level and task. Higher Learning Research Communications.

[B67-behavsci-16-00902] Sun P., Li J., Chen X., Cui M., Yan L. (2026). The impact of maladaptive perfectionism on AI dependence among college students: The mediating role of self-control and the moderating role of academic engagement. Acta Psychologica.

[B68-behavsci-16-00902] Tamrin S. I., Omar N. F., Kamaruzaman K. N., Zaghlol A. K., Abdul Aziz M. R. (2024). Evaluating the impact of AI dependency on cognitive ability among Generation Z in higher educational institutions: A conceptual framework. Information Management and Business Review.

[B69-behavsci-16-00902] Tangney J. P., Baumeister R. F., Boone A. L. (2004). High self-control predicts good adjustment, less pathology, better grades, and interpersonal success. Journal of Personality.

[B70-behavsci-16-00902] Thomas A. R. (1976). The organizational climate of schools. International Review of Education.

[B71-behavsci-16-00902] Usher E. L., Pajares F. (2008). Sources of self-efficacy in school: Critical review of the literature and future directions. Review of Educational Research.

[B72-behavsci-16-00902] Uyar A., Karafil B., Karakuyu A. (2026). Artificial intelligence dependency among educators: A scale development and validation study. Education and Information Technologies.

[B73-behavsci-16-00902] van Zyl L. E., Klibert J., Shankland R., See-To E. W. K., Rothmann S. (2022). The general academic self-efficacy scale: Psychometric properties, longitudinal invariance, and criterion validity. Journal of Psychoeducational Assessment.

[B74-behavsci-16-00902] Vieriu A. M., Petrea G. (2025). The impact of artificial intelligence (AI) on students’ academic development. Education Sciences.

[B75-behavsci-16-00902] Von Garrel J., Mayer J. (2023). Artificial Intelligence in studies—Use of ChatGPT and AI-based tools among students in Germany. Humanities and Social Sciences Communications.

[B76-behavsci-16-00902] Wang Q., Lee K. C. S., Hoque K. E. (2020). The effect of classroom climate on academic motivation mediated by academic self-efficacy in a higher education institute in China. International Journal of Learning, Teaching and Educational Research.

[B77-behavsci-16-00902] Wang S., Huang Y. (2024). Promote or inhibit: The impact of generative artificial intelligence on the creativity of college students. China Higher Education Research.

[B78-behavsci-16-00902] Wang Y., Wang L., Yang L., Wang W. (2024). Influence of perceived social support and academic self-efficacy on teacher-student relationships and learning engagement for enhanced didactical outcomes. Scientific Reports.

[B79-behavsci-16-00902] Wang Y., Xu S. (2026). Relationship between artificial intelligence tool usage experience and academic stress among college students: Mediating role of loneliness and moderating role of academic self-efficacy. Acta Psychologica.

[B80-behavsci-16-00902] Wei B., Zhuo Y., Zeng H., Hong H., Liu H. (2025). Determinants of university students’ attitudes towards smart devices in the smart campus environment. Humanities and Social Sciences Communications.

[B81-behavsci-16-00902] Wood R., Bandura A. (1989). Social cognitive theory of organizational management. The Academy of Management Review.

[B82-behavsci-16-00902] Xu J., Tian M., Hu D. (2026). Development and application of the generative artificial intelligence dependency scale for teachers. Journal of Distance Education.

[B83-behavsci-16-00902] Yang Y.-D., Zhou C.-L., Wang Z.-Q. (2024). The relationship between self-control and learning engagement among Chinese college students: The chain mediating roles of resilience and positive emotions. Frontiers in Psychology.

[B84-behavsci-16-00902] Yang Z., Deng H., Jiang N. (2025). The impact mechanism of artificial intelligence dependence on college students’ innovation capability: An empirical study from China. Frontiers in Psychology.

[B85-behavsci-16-00902] Yildiz Durak H., Yahşi Sari H., Dilmaç B., Durak A. (2026). Social anxiety and self-control desire for social media users: The mediating roles of resilience and social media usage. Journal of Rational-Emotive & Cognitive Behavior Therapy.

[B86-behavsci-16-00902] Yue H., Li C., Liu M., Jin R., Bao H. (2020). Validity test of the theory of planned behavior in college students’ withdrawal from smartphone dependence. Current Psychology.

[B87-behavsci-16-00902] Zhang H., Pan J. (2026). Generative AI dependence in higher education: A PLS-SEM examination of Chinese and Malaysian teachers through the I-PACE model. Acta Psychologica.

[B88-behavsci-16-00902] Zhang L., Xu J. (2025). The paradox of self-efficacy and technological dependence: Unraveling generative AI’s impact on university students’ task completion. The Internet and Higher Education.

[B89-behavsci-16-00902] Zhang S., Zhao X., Zhou T., Kim J. H. (2024). Do you have AI dependency? The roles of academic self-efficacy, academic stress, and performance expectations on problematic AI usage behavior. International Journal of Educational Technology in Higher Education.

[B90-behavsci-16-00902] Zhao L., Huang W., Han B., Wu X. (2026). Classroom climate dimensions, self-efficacy, and music aesthetic literacy: A mediation study with Chinese non-music major college students. Frontiers in Psychology.

[B91-behavsci-16-00902] Zhao X., Wang H., Ma Z., Zhang L., Chang T. (2025). Smartphone addiction and academic procrastination among college students: A serial mediation model of self-control and academic self-efficacy. Frontiers in Psychiatry.

[B92-behavsci-16-00902] Zhou S., Qin L., Deng J., Zhe J., Jiang D., Long S., Wu Y. (2025). How classroom climate is linked to prosocial behavior in undergraduate students: A multilevel moderated mediation model of feedback expectation and self-monitoring. BMC Psychology.

